# Supplementary material for: Plasma protein profiling predicts cancer in patients with non-specific symptoms
Source: Nat Commun. 2025 Dec 29;17:151. doi: 10.1038/s41467-025-67688-3 (PMC12774938; doi:10.1038/s41467-025-67688-3)
Supplement: Supplementary file 1 — Supplementary Information [file 41467_2025_67688_MOESM1_ESM.pdf]

Supplementary Information

Plasma protein profiling predicts cancer in patients with non-specific symptoms

Fredrika Wannberg[1*], María Bueno Álvez[2*], Alvida Qvick[3*], Tamas Pongracz[1], Katherina Aguilera[1], Emma Adolfsson[4], Louise Essehorn[5], Max Gordon[6], Mathias Uhlén[2], Gisela Helenius[7], Viktoria Hjalmar[8], Mikael Åberg[9*], Axel Rosell[1,10*], Charlotte Thålin[1*†]

1. Department of Clinical Sciences, Danderyd Hospital, Division of Internal Medicine, Karolinska Institutet, Stockholm, Sweden
2. Science for Life Laboratory, Department of Protein Science, KTH Royal Institute of Technology, Stockholm, Sweden
3. Clinical Research Center, Faculty of Medicine and Health, Örebro University, Örebro, Sweden
4. Department of Obstetrics and Gynecology, Faculty of Medicine and Health, Örebro University, Örebro, Sweden
5. Division of Internal Medicine, Danderyd Hospital, Stockholm, Sweden
6. Department of Clinical Sciences, Danderyd Hospital, Division of Orthopedics, Karolinska Institutet, Stockholm, Sweden
7. ATMP-center, Skåne University Hospital, Lund, Sweden
8. Division of Specialist Medical Care, Diagnostic center, Danderyd Hospital, Stockholm, Sweden
9. Department of Medical Sciences, Clinical Chemistry and SciLifeLab Affinity Proteomics, Uppsala University, Uppsala, Sweden
10. Center for Hematology and Regenerative Medicine (HERM), NEO, Department of Medicine, Huddinge, Karolinska Institutet, Stockholm, Sweden

*Equal contribution
†Corresponding author:
Charlotte Thålin
charlotte.thalin@ki.se

Supplementary Table S1. Baseline characteristics of patients in MEDECA and ALLVOS according to outcome during follow-up.

	**MEDECA**			**ALLVOS**		
	**All patients (n=456)**	**No cancer detected (n=296)**	**Cancer detected (n=160)**	**All patients (n=238)**	**No cancer detected (n=203)**	**Cancer detected (n=35)**
Female sex	252 (55)	166 (56)	86 (54)	119 (50)	102 (50)	17 (49)
Age	71 (60–78)	70 (57–76)	74 (66–81)	72 (61–78)	71 (59–78)	74 (71–83)
BMI	25 (22–28)	25 (22–28)	25 (23–28)	24.8 (21–28)	24.9 (21–28)	23.6 (22–27)
Current smoking	54 (12)	33 (11)	21 (13)	39 (16)	34 (17)	5 (14)
Autoimmune disease[a]	78 (17)	59 (20)	19 (12)	12 (5)	11 (5)	1 (3)
COPD	47 (10)	32 (11)	15 (9)	15 (6)	11 (5)	4 (11)
DM	75 (16)	51 (17)	24 (15)	50 (21)	39 (19)	11 (31)
Arterial disease	76 (17)	47 (16)	29 (18)	36 (15)	31 (15)	5 (14)
Previous cancer	75 (16)	46 (16)	29 (18)	38 (16)	27 (13)	11 (31)

[a] Hypothyroidism not included; IQR, inter quartile range; BMI, body mass index; COPD, chronic obstructive pulmonary disease; DM, diabetes type 1 or type 2. Results are presented as number (%) or median (IQR)

Supplementary Table S2. Cancer types diagnosed during follow-up in MEDECA (discovery cohort) and ALLVOS (replication cohort). Results are presented as number (%).

	**MEDECA**		**ALLVOS**	
**Cancer site[a]**	**Cancer during follow-up (n = 160[b])**	**Metastases at diagnosis (n=102[b])**	**Cancer during follow-up (n = 35[b])**	**Metastases at diagnosis (n=12[b])**
Carcinoma	103 (64)	91 (88)	25 (71)	12 (48)
Germinal	2 (1.3)	2 (100)	0 (0)	0 (0)
Urothelial	4 (2.5)	3 (75)	0 (0)	0 (0)
Neuroendocrine tumor	12 (7.5)	9 (75)	3 (8.6)	1 (33)
Adenocarcinoma	80 (50)	71 (89)	17 (49)	8 (47)
Breast	9 (5.6)	8 (89)	2 (5.7)	0 (0)
HCC	6 (3.8)	4 (67)	0 (0)	0 (0)
Kidney	5 (3.1)	4 (80)	0 (0)	0 (0)
Colorectal	5 (3.1)	5 (100)	1 (2.9)	0 (0)
Prostate	4 (2.5)	2 (50)	1 (2.9)	0 (0.0)
Lung	13 (8.1)	11 (85)	2 (5,7)	1 (50)
Gastric	2 (1.3)	2 (100)	0 (0)	0 (0)
Thyroid	4 (2.5)	3 (75)	1 (2.9)	0 (0.0)
Ovaries and tubar	8 (5.0)	8 (100)	1 (2.9)	1 (100)
Pancreas, gall bladder and bile duct	17 (11)	17 (100)	2 (5.7)	1 (50)
Parotid	1 (0.6)	1 (100)	0 (0)	0 (0)
Appendix	3 (1.9)	3 (100)	1 (2.9)	0 (0)
Pseudomyxoma	1 (0.6)	1 (100)	0 (0)	0 (0)
Adenocarcinoma with unknown primary	2 (1.3)	2 (100)	3 (8.6)	3 (100)
Mesothelioma	3 (1.9)	3 (100)	0 (0)	0 (0)
Squamous cell carcinoma[c]	0	0	5 (14.3)	2 (40.0)
Carcinoma with unknown primary	3 (1.9)	3 (100)	1 (2.9)	1 (100.0)
Sarcoma	4 (2.5)	3 (75)	0 (0)	0 (0)
GIST	1 (0.6)	0 (0)	0 (0)	0 (0)
Other sarcoma	3 (1.9)	3 (100)	0 (0)	0 (0)
Myeloma	15 (9.4)	NA	1 (2.9)	NA
Lymphoma	24 (15.0)	NA	4 (11.4)	NA
B-cell-lymphoma	8 (5.0)	NA	1 (2.9)	NA
T-cell lymphoma	2 (1.3)	NA	0 (0)	NA
Follicular lymphoma	4 (2.5)	NA	1 (2.9)	NA
Hodgkin’s lymphoma	5 (3.1)	NA	1 (2.9)	NA
Mantle cell lymphoma	2 (1.3)	NA	0 (0)	NA
Waldenstrom macroglobulinemia	1 (0.6)	NA	0 (0)	NA
Chronic lymphocytic leukemia	2 (1.3)	NA	1 (2.9)	NA
MDS	5 (3.1)	NA	1 (2.9)	NA
Acute myeloid leukemia	0	NA	1 (2.9)	NA
Myeloproliferative neoplasm	1 (0.6)	NA	0 (0)	0 (0)
Melanoma	0 (0)	0 (0)	0 (0)	0 (0)
Glioblastoma	1 (0.6)	1 (100)	0 (0)	0 (0)
Cancer with unknown primary	7 (4.4)	7 (100)	4 (11.4)	0 (0.0)

GIST, gastrointestinal stromal tumor; MDS, myelodysplastic syndrome; NA; Not applicable.
[a] Categorized according to Swedish national cancer care program for cancers without known primary (1, 2) [b] The total number of carcinomas exceeds 103 and 25 respectively, as one patient in each cohort had more than one primary tumor [c] All located in lung

Supplementary Table S3 Non-malignant diagnoses among patients without cancer during follow-up in MEDECA (n=155)

**Diagnosis**	**Number of patients**[a]
Infections	32
Bacterial	24
Viral	6
Fungal	2
Parodontitis	7
Autoimmune disease	55
Polymyalgia rheumatica	8
Giant cell arteritis/vasculitis<br>Microscopic polyangiitis	12<br>2
Rheumatoid arthritis/polyarthritis/monoarthritis	8
Sarcoidosis	7
Primary biliary cholangitis	3
Autoimmune hepatitis	2
Psoriatic arthritis	2
Systemic lupus erythematosus	2
Retroperitoneal fibrosis	2
Alcohol dependency	8
Gastrointestinal inflammation (Barrets, gastritis, duodenitis and diverticulitis)	6
Osteoporosis with or without pathological fractures	6
Pericardial effusion/non-infectious percarditis	3
Heart failure	3
Liver cirrhosis	3
Venous thromboembolism	4
Ureter/kidney stone	2
Alcoholic liver disease (no cirrhosis)	2
Iron deficiency due to gastrointestinal bleeding	2

List of diagnoses that occurred once. Autoimmune diseases: Autoimmune pancreatitis, celiac disease, Grave's disease, myositis, morphea, polyarteritis nodosa, psoriasis, Paget's disease of bone, pelvospondylitis, Sjögren's syndrome, systemic inflammatory disease unspecified[b]. Other diagnoses: aseptic lymphocyte-dominant vasculitis-associated lesion, acute tubulointerstitial nephritis caused by Asacol, bursitis, chronic osteomyelitis, diabetes mellitus type 2, endometriosis, femoral head necrosis, gout, hidradenitis suppurativa, hypersensitivity pneumonitis, inflammation in thigh of unknown cause, kidney failure, Langerhans' cell histiocytosis, liver cysts with inferior vena cava compression, lumbar disc herniation with reactive lymph nodes, meningioma, necrotizing granuloma of the liver of unknown cause, pancreatic insufficiency, pleural effusion, polyarthrosis, polycystic ovaries, primary hyperparathyroidism, rectal adenoma with high grade dysplasia, Riedel thyroiditis, small bowel ischemia, spinal hemangioma, substance abuse (anabolic steroids), Wernicke encephalopathy.
[a]The total number of diagnoses (165) exceeds 155 as some patients received more than one diagnosis.
[b]High erythrocyte sedimentation rate was treated with Methotrexate

Supplementary Table S4 Non-malignant diagnoses among patients without cancer during follow-up in ALLVOS (n=120)

**Diagnosis**	**Number of patients**[a]
Infections	23
Bacterial	18
Viral	5
Autoimmune disease	26
Polymyalgia rheumatica	15
Celiac disease	2
Giant cell arteritis/vasculitis	2
Sarcoidosis	2
Myositis	2
Alcohol dependency	6
Gastrointestinal inflammation (ulcus, gastritis, colitis)	12
Arthrosis	3
Heart failure	3
Kidney failure	2
Diabetes mellitus type 2	2
Chronic obstructive pulmonary disease	2
Iron deficiency anemia due to gastrointestinal bleeding[b]	5
Iron deficiency anemia of unspecified cause	4
Drug side effects	8

List of diagnoses that occurred once. Autoimmune diseases: Arteritis, autoimmune hepatitis, primary biliary cholangitis, small vessel vasculitis. Other diagnoses: non-toxic goiter, gout, inflammation due to foreign body in colon, panniculitis, systemic inflammatory disease unspecified[c], colon adenoma, asthma, intermittent claudication, fibromyalgia, hiatus hernia, shoulder impingement, intraosseous meningioma, liver cirrhosis, B12-deficience due to malnutrition and malabsorption, menopause, nephrotic syndrome, osteomalacia, portal hypertensive gastropathy, testosterone deficiency.

[a]The total number of diagnoses (166) exceeds 120 as some patients received more than one diagnosis.

[b]One patient with clinical suspicion of GI-bleeding

[c]Treated with systemic steroids

Supplementary Table S5

Results from logistic regression models adjusted for age and sex for the 22 differentially expressed proteins. Odds ratios (ORs), standard error (SE), 95% confidence intervals (CIs), and corresponding raw and adjusted (Benjamini-Hochberg correction) p-values are reported.

**Cohort**	**Assay**	**OR**	**SE**	**Statistic**	***P* value**	**Adjusted *P* value**	**CI low**	**CI high**
Discovery	RRM2	2.39	0.12	7.23	4.74e-13	4.63e-10	1.91	3.07
Discovery	PARP1	3.06	0.15	7.26	3.81e-13	4.46e-10	2.3	4.21
Discovery	PSIP1	2.28	0.12	7.07	1.55e-12	1.30e-09	1.84	2.91
Discovery	KRT19	1.83	0.08	7.78	7.51e-15	4.39e-11	1.58	2.14
Discovery	DPY30	2.01	0.09	7.51	5.79e-14	1.70e-10	1.69	2.44
Discovery	MAD1L1	2.36	0.12	7.46	8.97e-14	1.75e-10	1.9	2.99
Discovery	KRT18	1.7	0.07	7.28	3.28e-13	4.46e-10	1.48	1.97
Discovery	ELOA	2.53	0.14	6.87	6.46e-12	4.20e-09	1.97	3.34
Discovery	ZBTB17	2.39	0.13	6.77	1.33e-11	6.00e-09	1.88	3.11
Discovery	AGR2	1.54	0.06	6.77	1.33e-11	6.00e-09	1.36	1.75
Discovery	GRPEL1	1.62	0.07	6.82	9.16e-12	5.36e-09	1.42	1.87
Discovery	FKBP4	2.73	0.15	6.91	5.00e-12	3.66e-09	2.07	3.66
Discovery	STC1	2.45	0.13	6.75	1.47e-11	6.16e-09	1.91	3.21
Discovery	APEX1	2.16	0.12	6.3	2.90e-10	6.52e-08	1.71	2.77
Discovery	METAP1D	1.76	0.09	6.16	7.41e-10	1.45e-07	1.48	2.12
Discovery	PRDX3	1.82	0.1	6.09	1.16e-09	2.14e-07	1.51	2.22
Discovery	PAEP	1.38	0.05	6.16	7.37e-10	1.45e-07	1.25	1.53
Discovery	EIF4G1	1.73	0.09	6.35	2.22e-10	5.19e-08	1.47	2.06
Discovery	PTS	1.57	0.08	5.79	6.98e-09	8.88e-07	1.36	1.85
Discovery	HDGF	1.63	0.09	5.27	1.34e-07	8.80e-06	1.36	1.96
Discovery	BAIAP2	1.54	0.08	5.14	2.71e-07	1.34e-05	1.31	1.83
Discovery	S100A12	1.27	0.06	3.72	1.97e-04	5.13e-04	1.12	1.43
Replication	RRM2	2.86	0.22	4.75	2.02e-06	5.11e-04	1.93	4.61
Replication	PARP1	3.2	0.24	4.94	7.79e-07	5.11e-04	2.06	5.21
Replication	PSIP1	2.84	0.2	5.26	1.44e-07	4.21e-04	1.96	4.3
Replication	KRT19	1.97	0.14	4.74	2.09e-06	5.11e-04	1.51	2.65
Replication	DPY30	2.89	0.21	5.1	3.41e-07	5.11e-04	1.97	4.47
Replication	MAD1L1	5.57	0.3	5.77	8.08e-09	4.73e-05	3.25	10.5
Replication	KRT18	1.58	0.14	3.2	1.35e-03	5.29e-03	1.19	2.1
Replication	ELOA	2.83	0.23	4.44	8.94e-06	5.11e-04	1.82	4.6
Replication	ZBTB17	4	0.3	4.63	3.70e-06	5.11e-04	2.28	7.44
Replication	AGR2	1.42	0.13	2.73	6.41e-03	1.49e-02	1.1	1.83
Replication	GRPEL1	2.33	0.18	4.76	1.97e-06	5.11e-04	1.67	3.37
Replication	FKBP4	3.7	0.31	4.26	2.06e-05	5.11e-04	2.09	7.01
Replication	STC1	2.64	0.26	3.71	2.03e-04	1.02e-03	1.61	4.51
Replication	APEX1	2.31	0.21	4.08	4.51e-05	5.11e-04	1.56	3.51
Replication	METAP1D	2.09	0.18	4.11	3.92e-05	5.11e-04	1.49	3.03
Replication	PRDX3	1.94	0.18	3.7	2.20e-04	1.08e-03	1.38	2.8
Replication	PAEP	1.37	0.11	2.93	3.34e-03	1.09e-02	1.11	1.69
Replication	EIF4G1	1.53	0.14	3	2.74e-03	9.66e-03	1.17	2.05
Replication	PTS	1.84	0.19	3.16	1.58e-03	6.08e-03	1.28	2.72
Replication	HDGF	1.79	0.16	3.52	4.36e-04	1.92e-03	1.31	2.5
Replication	BAIAP2	1.9	0.2	3.28	1.06e-03	4.22e-03	1.3	2.83
Replication	S100A12	1.45	0.11	3.29	1.01e-03	4.05e-03	1.16	1.81

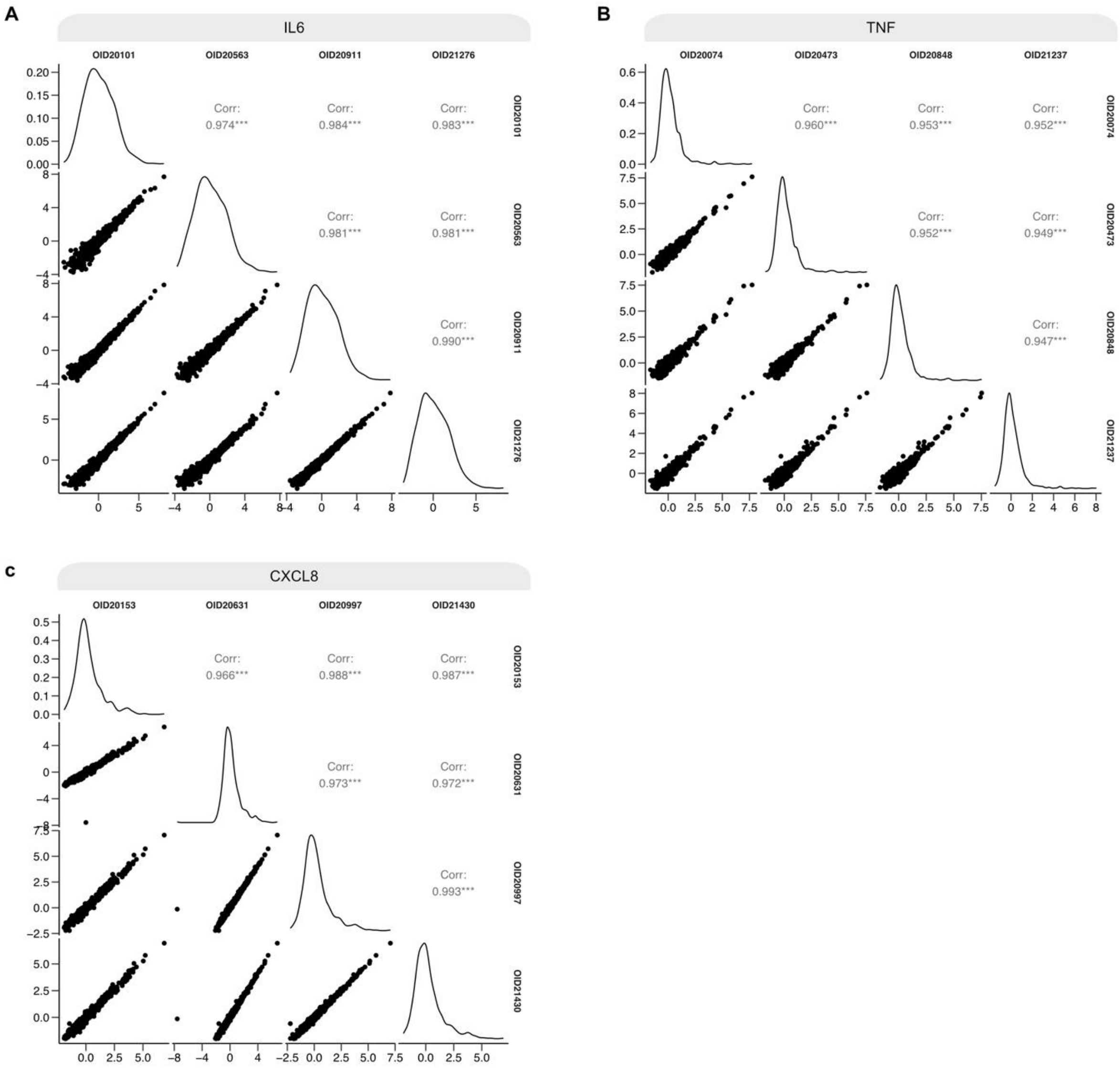

Supplementary Figure S1. Correlation between replicate assays in the Olink data for **A)** IL6, **B)** TNF and **C)** CXCL8. Correlogram generated using the ggpairs function (GGally package) showing pairwise relationships among replicate protein abundances across all samples (n = 808 samples) for each protein. Upper panels display the Pearson correlation coefficients (r) and associated unadjusted two-sided p-values (*** = p<0.001). Lower panels show pairwise scatterplots with individual data points, and the diagonal panels show density plots representing the distribution of protein abundance in NPX unit.

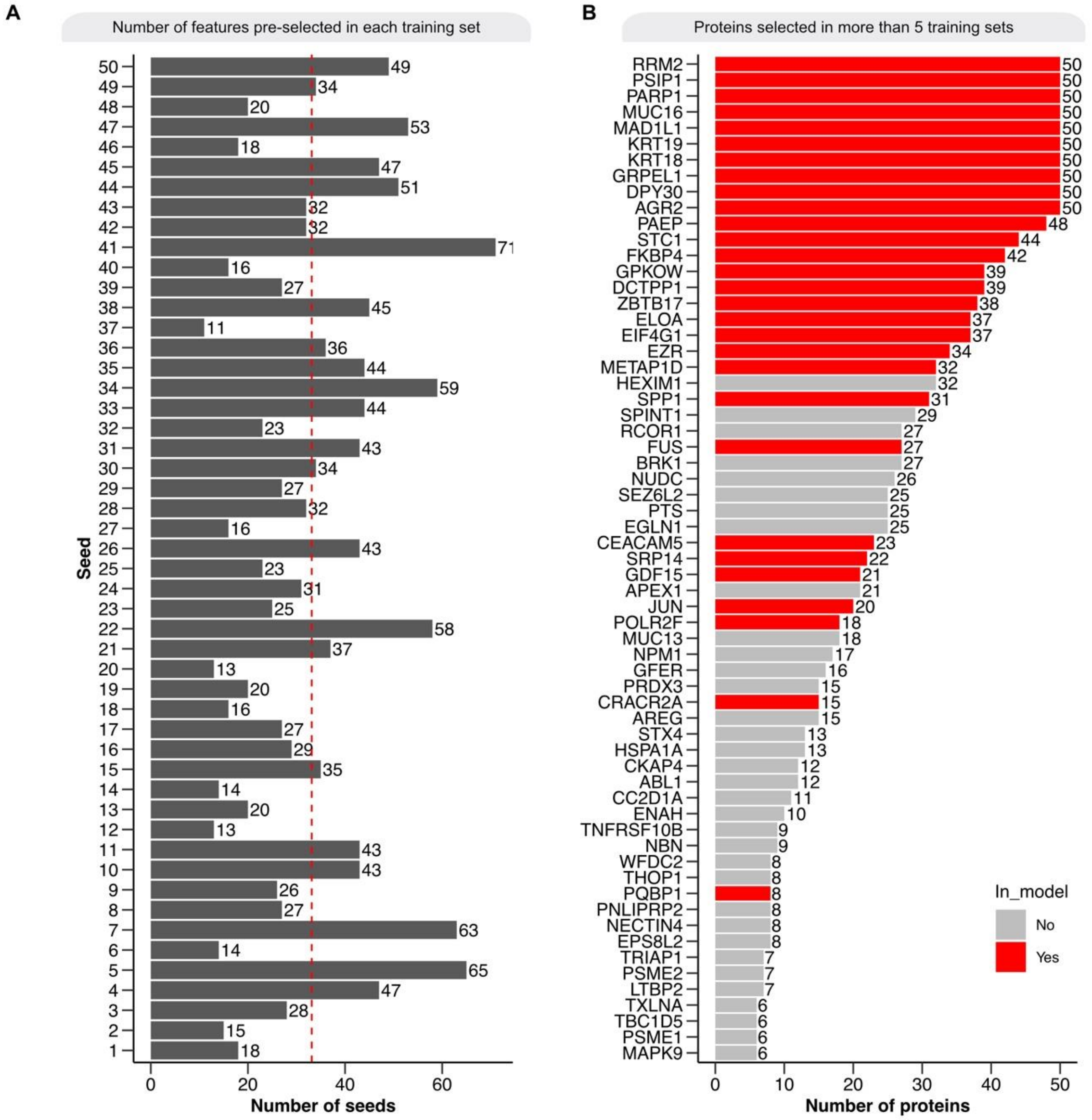

Supplementary Figure S2. Stability of the cancer classification model in MEDECA. A) Number of proteins pre-selected after univariate filtering in 50 generated training sets. The dashed line is the mean number of proteins. B) Number of times each protein was selected.

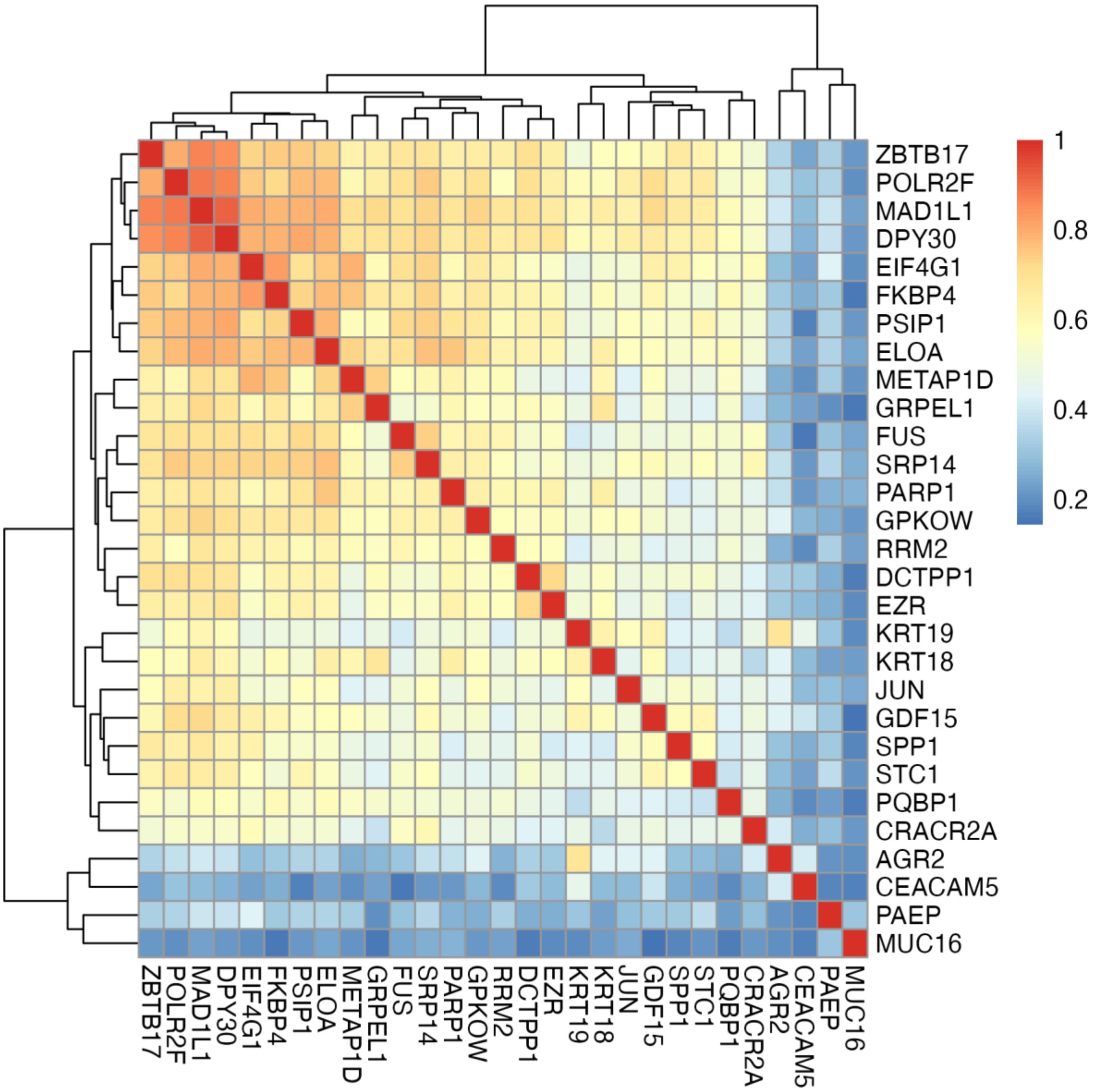

Supplementary Figure S3. Correlation (Spearman) between selected proteins in the final model.

References

1. Sweden RCC. Cancer utan känd primärtumör, CUP, Nationellt vårdprogram. 2021.
2. Lin F, Liu H. Immunohistochemistry in undifferentiated neoplasm/tumor of uncertain origin. Arch Pathol Lab Med. 2014;138(12):1583-610.
